# A Modified Constitutive Model and Microstructure Characterization for 2195 Al-Li Alloy Hot Extrusion

**DOI:** 10.3390/ma16103826

**Published:** 2023-05-18

**Authors:** Hui Li, Jian Wang, Yuanchun Huang, Rong Fu

**Affiliations:** 1 College of Mechanical and Electrical Engineering, Central South University, Changsha 410083, China; lih0605@163.com (H.L.);; 2 State Key Laboratory of High-Performance Complex Manufacturing, Central South University, Changsha 410083, China; 3 Southwest Aluminum (Group) Co., Ltd., Chongqing 400050, China; 4 Light Alloy Research Institute, Central South University, Changsha 410083, China

**Keywords:** 2195 Al-Li alloy, constitutive model, extrusion numerical simulation, microstructure

## Abstract

The quality of extruded profiles depends largely on accurate constitutive models and thermal processing maps. In this study, a modified Arrhenius constitutive model for homogenized 2195 Al-Li alloy with multi-parameter co-compensation was developed and further enhanced the prediction accuracy of flow stresses. Through the processing map and microstructure characterization, the 2195 Al-Li alloy could be deformed optimally at the temperature range of 710~783 K and strain rate of 0.001~0.12 s^−1^, preventing the occurrence of local plastic flow and abnormal growth of recrystallized grains. The accuracy of the constitutive model was verified through numerical simulation of 2195 Al-Li alloy extruded profiles with large shaped cross-sections. Dynamic recrystallization occurred at different regions during the practical extrusion process, resulting in slight variations in the microstructure. The differences in microstructure were due to the varying degrees of temperature and stress experienced by the material in different regions.

## 1. Introduction

Compared with ordinary aluminum alloys, Al-Li alloy components possess lighter weight, higher mechanical properties and fatigue resistance [1,2]. These features make them popular in rail transportation, aircraft, and other industries. Hot extrusion is capable of producing complex shapes with high dimensional accuracy and good mechanical properties, and has become one of the important forming processes [3].

The accuracy of extrusion numerical simulation and the consistency of profiles depend heavily on an accurate constitutive model. Consequently, different types of constitutive models have been developed and have gained some applications. Jonas et al. [4] first established an Arrhenius hyperbolic sinusoidal constitutive model to describe the thermal deformation behavior of alloys. However, this model only considers the effects of deformation temperature and strain rate on flow stress. Therefore, Lin et al. [5] modified the conventional Arrhenius constitutive model using a strain-strain rate compensation method to increase the accuracy of flow stress predictions over wide ranges of strain rate and forming temperature. Subsequently, a new phenomenological model was proposed [6], where the material constants were expressed as functions of strain rate and temperature, enabling the accurate prediction of flow stress in the Al-Cu-Mg alloy. Additionally, a strain-compensated Arrhenius constitutive model was developed by some researchers in order to accurately predict high-temperature flow stresses in different grades of aluminum alloys [7], magnesium alloys [8], steel [9], titanium alloys [10], and other materials. Chen et al. [11] compensated the strain rate and deformation temperature based on the strain-compensated Arrhenius constitutive model, further improving the prediction accuracy.

The work hardening and dynamic softening coincide in the high-temperature rheological process of aluminum alloy, where dynamic recovery (DRV) and dynamic recrystallization (DRX) are the main softening mechanisms. The microstructure of alloys is usually affected by the thermal deformation parameters, and the specific microstructure formed after deformation is the main factor leading to the difference in material properties. The processing map based on the dynamic materials model (DMM) proposed by Prasad et al. [12] can reflect the relationship between the microstructure evolution mechanism and the thermal deformation parameters; therefore a lot of research has been conducted in this field [7,11,13]. Zhang et al. [14] investigated the DRX behavior of 2195 Al-Li alloy at moderate/high temperatures, they evaluated that the primary softening mechanism for deformation at moderate temperatures is discontinuous dynamic recrystallization (DDRX), and it transforms to continuous dynamic recrystallization (CDRX) at high temperatures. Yu et al. [15] found that 2A97 Al-Li alloy exhibited the most elevated power dissipation efficiency in the DRX region using processing maps and indicated that the occurrence of CDRX was related to the dynamic precipitation of the T_1_ phase. Wang et al. [16] discovered that the spray-deposited 2195 Al-Li alloy produces high power dissipation efficiency at low temperature and low strain rate, while the lowest power dissipation efficiency was observed at a strain rate of 10 s^−1^ and the material undergoes deformation instability. During thermal deformation, alloys with the same composition in various states will exhibit considerably variable power dissipation efficiencies. Therefore, it is necessary to study the effect of alloy state on the evolution of microstructure.

Currently, significant progress has been made in researching the hot deformation behavior of aluminum alloys. However, there are few successful cases where the obtained constitutive model and hot processing map have been applied to actual industrial production, particularly for Al-Li alloys. Dong et al. [17] simulated the extrusion process of complex cross-section profile by establishing the Arrhenius constitutive model and processing map of AA6N01 aluminum alloy and effectively controlled the distortion and deformation. Xu et al. [18] revealed the cause of abnormal grain growth along the longitudinal weld of the profile in 2196 Al-Cu-Li alloy by finite element method. Zhang et al. [19] used HyperXtrude to build a numerical model of the transverse welds of 7N01 aluminum alloy, and by adjusting the extrusion ratio and die structure, the length of the transverse welds was effectively reduced and high-quality extruded profiles were obtained. In this study, hot compression tests were conducted on the two-stage homogenized 2195 Al-Li alloy, and the modified Arrhenius constitutive model as well as the processing map were established. Then, numerical simulations were carried out using HyperXtrude software to analyze the flow behavior of large shaped cross-sectional profiles of 2195 Al-Li alloy based on the established constitutive model. Finally, practical engineering extrusion tests were conducted and the microstructure in different regions was examined to validate the accuracy of the constitutive model and simulation results. The research strategy of constitutive model, processing map establishment—numerical simulation—actual extrusion test—microstructure analysis will provide strong support for the large-scale industrial production of 2195 Al-Li alloy profiles.

## 2. Experimental Materials and Methods

### 2.1. Material and Hot Compression Tests

The 2195 Al-Li Alloy was provided by Southwest Aluminum (Group) Corporation of China Ltd. (Chongqing, China), and the chemical composition of the alloy is 3.98Cu, 0.99Li, 0.32Mg, 0.32Ag, 0.12Zr, 0.08Fe, 0.04Si (wt%) and a balance of Al. The material was sampled at the center of the ingot after a two-stage homogenization treatment of 470 °C/7 h + 525 °C/24 h and machined into a hot compression specimen of Ø8 × 12 mm for diameter and height. Hot compression tests were performed on the Gleeble-3500D thermomechanical simulator with a strain rate range of 0.001–1 s^−1^, a 60% height depression, and a deformation temperature range of 643–693 K. The specimen was first heated to the required temperature at a rate of 5 K/s, held there for 3 min during the test, and then hot-compressed at a specific strain rate. Finally, water-cooled quenching was applied immediately after reaching the required reduction to preserve the high-temperature microstructure. Before the hot compression experimental process, graphite foils were placed between the upper and lower ends of the specimen and the contact surface of the WC anvil to reduce friction.

### 2.2. Establishment of Extrusion Assembly

**Figure 1:**
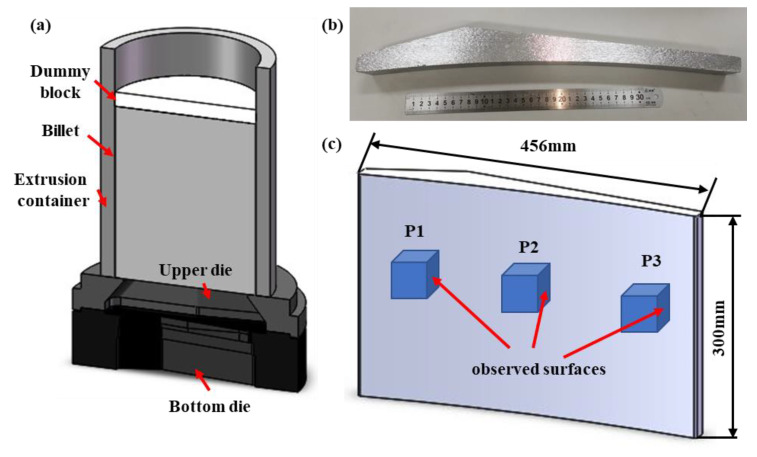
(**a**) Extrusion modeling of assembly diagram, (**b**) The practical 2195 Al-Li alloy extruded profile, (**c**) sampling positions of the profile.

Figure 1a displays the extrusion assembly diagram of the geometric model, which consists of four components, namely the extrusion container, dummy block, billet and extrusion die. Relevant extrusion process parameters are detailed in Table 1.

### 2.3. Microstructure Observation

The as-cast and homogenized microstructures were observed by metallographic microscope (OLYMPOS-DSX500) and scanning electron microscope (SEM, JSM-7800F), respectively, and the chemical composition of the second phase was analyzed by energy dispersive spectrometer (EDS, Xmax-80). In addition, the composition and type of phases contained in the two alloys were identified by X-ray diffractometry (D8 Advance). The microstructural characterization of hot-compressed samples with different process parameters and profiles in different regions after the actual engineering extrusion was carried out using electron backscatter diffraction (EBSD) to investigate the deformation mechanism behind them, which was performed at the Helios Nanolab 600i electron microscope. The sampling position of the hot compressed samples was located in the central area along the compression axis, and the profile was divided into three regions: P1, P2 and P3. The practical 2195 Al-Li alloy extruded profile is shown in Figure 1b, and the observed surfaces are shown in Figure 1c. The specimens used for EBSD analysis were water-ground, mechanically polished, and then placed in electrolytic polishing solutions (90% C_2_H_5_OH + 10% HClO_4_) for 5–8 s at room temperature using the parameter of voltage 20 V. The collected EBSD data were analyzed and processed using HKL Channel-5 software.

## 3. Results and Discussion

### 3.1. Initial Microstructure

**Figure 2:**
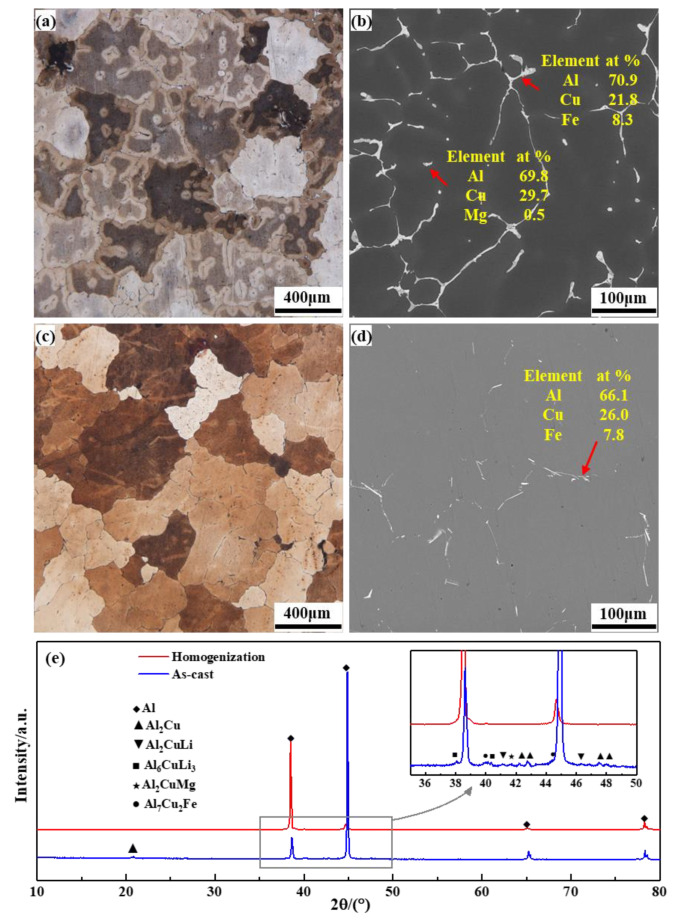
OM, SEM images and XRD pattern of the as-cast and homogenized 2195 Al-Li alloy: (**a**,**b**) as-cast, (**c**,**d**) homogenized, (**e**) XRD pattern.

The OM, SEM and XRD diagrams of the as-cast and homogenized 2195 Al-Li alloy are shown in Figure 2. The as-cast alloy shown in Figure 2a has severe dendritic segregation and a sizeable amount of continuously scattered non-equilibrium eutectic organization near to the grain boundary. According to Figure 2b, skeleton coarse second phases are continually dispersed along grain boundaries, whereas a small number of slate-like second phases are scattered throughout the grain. According to the results of the separate EDS analysis of the special second phase’s composition, the slate-like second phase is the Al_2_Cu phase, while the skeleton second phase is primarily made up of Al, Cu, and Fe elements. Following homogenization, as seen in Figure 2c,d, dendritic segregation is eliminated, leaving only a few sporadic second phases in the matrix. According to the EDS data, these second phases are primarily Al_7_Cu_2_Fe phases. Based on the results of the XRD analysis of the as-cast and homogenized states shown in Figure 2e, the ingot still contains some T_1_(Al_2_CuLi), T_2_(Al_6_CuLi_3_) and S(Al_2_CuMg) phases. However, after homogenization, these phases are essentially redissolved into the aluminum matrix.

### 3.2. Flow Stress Behavior

**Figure 3:**
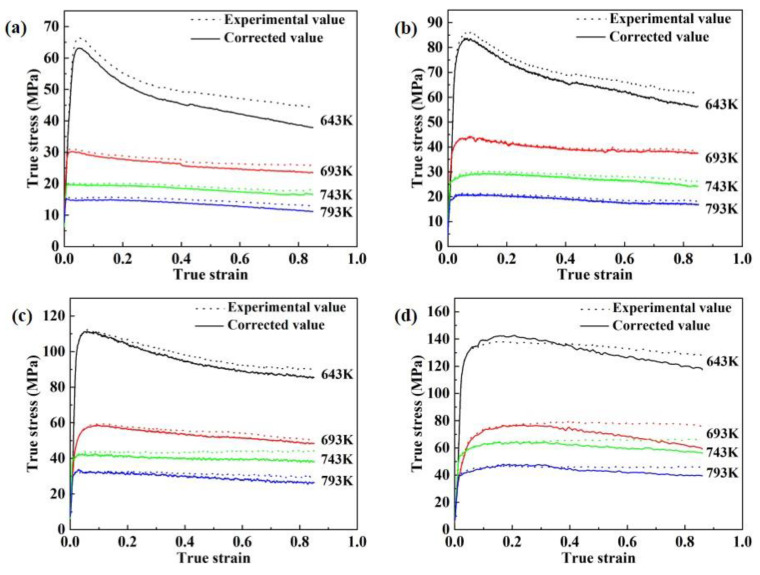
Comparisons of stress-strain curves of 2195 Al-Li alloy before and after friction and temperature corrections: (**a**) 0.001 s^−1^, (**b**) 0.01 s^−1^, (**c**) 0.1 s^−1^, (**d**) 1 s^−1^.

The true stress-strain curves of the two-stage homogenized 2195 Al-Li alloy obtained by isothermal hot compression tests at different deformation conditions are shown in Figure 3, and the friction and temperature corrections were applied using the method proposed by Ebrahimi and Najafizadeh [20]. As observed in Figure 3, the corrected flow stresses are all smaller than the uncorrected flow stresses, and the average absolute relative error is 6.96%, indicating that the friction between the specimen end face and the indenter increases the real load. It can be seen from the corrected flow stress curves that the flow stress of 2195 Al-Li alloy is significantly affected by the strain rate, deformation temperature and strain.

At the initial stage of deformation, the stress rises rapidly with the increase in strain, and then decreases gradually when the stress reaches the peak, and finally stabilizes. At the fixed deformation temperature, the stress rises with the increase in strain rate because the dislocations caused by the rapid deformation are not ready to eliminate and rearrange in a short time, which leads to an increase in the work hardening rate. Due to the thermal activation of metal atoms being strengthened at higher temperatures and the metal’s deformation resistance declining, the stress reduces with increasing deformation temperature at the same strain rate. Additionally, DRV and DRX tend to occur when the alloy is deformed at high temperatures, resulting in alloy softening [21,22].

### 3.3. Establishment of the Constitutive Model

#### 3.3.1. Arrhenius Constitutive Model with Strain Compensation

The Arrhenius hyperbolic sinusoidal constitutive equation is typically used to depict the connection between flow stress, deformation temperature, and strain rate of metal or alloy during thermal deformation, that is:

(1) $$\dot{\varepsilon }=AF(\sigma )\exp(-\frac{Q}{RT})$$

(2) $$F(\sigma )=\left\{\begin{array}{c} \sigma^{n_{1}}\\ \exp(\beta \sigma )\\ {\left[\sinh(\alpha \sigma )\right]}^{n} \end{array}\right.\begin{array}{c} \;(\alpha \sigma <0.8)\\ \;(\alpha \sigma >1.2)\\ \;(\mathrm{for}\;\mathrm{all}\;\sigma ) \end{array}$$

where $\dot{\varepsilon }$ is the strain rate (s^−1^), *σ* is the flow stress (MPa), *R* is the universal gas constant (8.314 J mol^−1^ K^−1^), *T* is the thermodynamic temperature (K), *Q* is the deformation thermal activation energy (kJ mol^−1^), and *A, α, n*_1_*, n, and β* are material constants, where *α = β/n*_1_. To calculate the value of each constant, Equation (2) is brought into Equation (1) on each side and the logarithm taken to obtain:

(3) $$\ln\dot{\varepsilon }=\left\{\begin{array}{c} \ln A_{1}+n_{1}\ln\sigma -\frac{Q}{RT}\;(\alpha \sigma <0.8)\\ \ln A_{2}+\beta \sigma -\frac{Q}{RT}\;(\alpha \sigma >1.2)\\ \ln A_{3}+n\ln[\sinh(\alpha \sigma )]-\frac{Q}{RT}\;(\mathrm{for}\;\mathrm{all}\;\sigma ) \end{array}\right.$$

**Figure 4:**
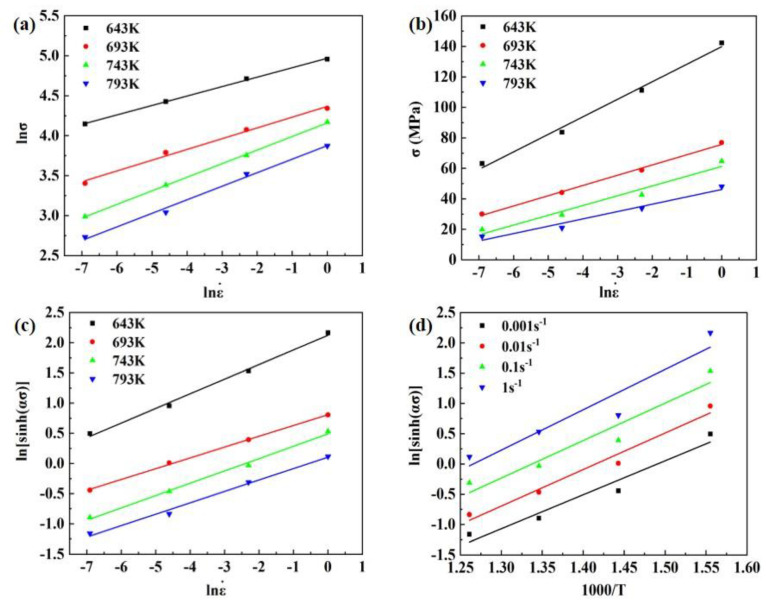
Fitting line of different deformation rate intervals: (**a**) $\ln\dot{\;\varepsilon }\;–\;\ln\;\sigma$, (**b**) $\ln\dot{\;\varepsilon }\;–\;\sigma$, (**c**) $\ln\;[sinh\left(\alpha \sigma \right)]-\ln\dot{\;\varepsilon }$, (**d**) $\ln\;[sinh\left(\alpha \sigma \right)]-T^{-1}$.

The solutions for each parameter in this experiment are used for peak stresses with different deformation conditions, and the specific data are shown in Table 2. The relationship between $\ln\dot{\varepsilon }-\ln\sigma$ and $\ln\dot{\varepsilon }-\sigma$ obtained is shown in Figure 4a,b based on the experimental data, and the values of *n*_1_ and *β* can be regressed to 6.7498 and 0.1357 using the linear fitting method, respectively, then *α = β/n*_1_ = 0.0201.

For all stress states, combining Equations (1) and (2), and taking partial derivatives on both sides gives:

(4) $$Q=R{\left\{\frac{\partial \ln\dot{\varepsilon }}{\partial \ln[\sinh(\alpha \sigma )]}\right\}}_{T}{\left\{\frac{\partial \ln[\sinh(\alpha \sigma )]}{\partial (1/T)}\right\}}_{\dot{\varepsilon }}$$

Based on the peak stress value, the functional relationship between $\ln[\sinh(\alpha \sigma )]-\ln\dot{\varepsilon }$ and $\ln[\sinh(\alpha \sigma )]-T^{-1}$ can be obtained, as shown in Figure 4c,d. A linear fit to the data in Figure 4c yields *n* = 4.9057. According to the obtained *n* and the average value of *Q/nR* obtained by linear fitting the data in Figure 4d, the average value of *Q* can be calculated as 249.722 kJ mol^−1^. Based on obtaining *n* and *Q*, combined with Figure 4c, the average value of *A* can be obtained as 2.5052 × 10^16^. Based on the fitted material parameters, the isothermal hot compression constitutive equation is obtained as shown in Equation (5).

(5) $$\dot{\varepsilon }=2.5052\times 10^{16}{\left[\sinh(0.0201\sigma )\right]}^{4.9057}\exp\left[-\frac{246386}{RT}\right]$$

Since the strain rate is controlled by the thermal activation energy and temperature during plastic deformation, this coupling relationship can be expressed by the Zener-Hollomon coefficient, which can be rewritten as:

(6) $$Z=\dot{\varepsilon }\exp(\frac{Q}{RT})=A{\left[\sinh(\alpha \sigma )\right]}^{n}$$

The expression for the flow stress containing the Zener-Hollomon coefficient can be obtained from the definition of the hyperbolic sine function as follows:

(7) $$\sigma =\frac{1}{\alpha }\ln\left\{{\left(\frac{Z}{A}\right)}^{1/n}+{\left[{\left(\frac{Z}{A}\right)}^{2/n}+1\right]}^{1/2}\right\}$$

The calculated data are brought to the hyperbolic sine function with the Zener-Hollomon coefficient at peak stress, which can be expressed as:

(8) $$\sigma =49.7193\ln\left\{{\left(\frac{Z}{2{.5052\times 10}^{16}}\right)}^{0.2038}+{\left[{\left(\frac{Z}{2{.5052\times 10}^{16}}\right)}^{0.4077}+1\right]}^{1/2}\right\}$$

The Arrhenius model previously established is a function of flow stress on deformation temperature and deformation rate, and does not consider the influence of deformation degree on flow stress. In order to more accurately predict the flow stresses in the alloy during the hot compression process, it is necessary to establish a strain-compensated Arrhenius constitutive model that considers the deformation temperature, deformation rate and deformation degree simultaneously.

**Figure 5:**
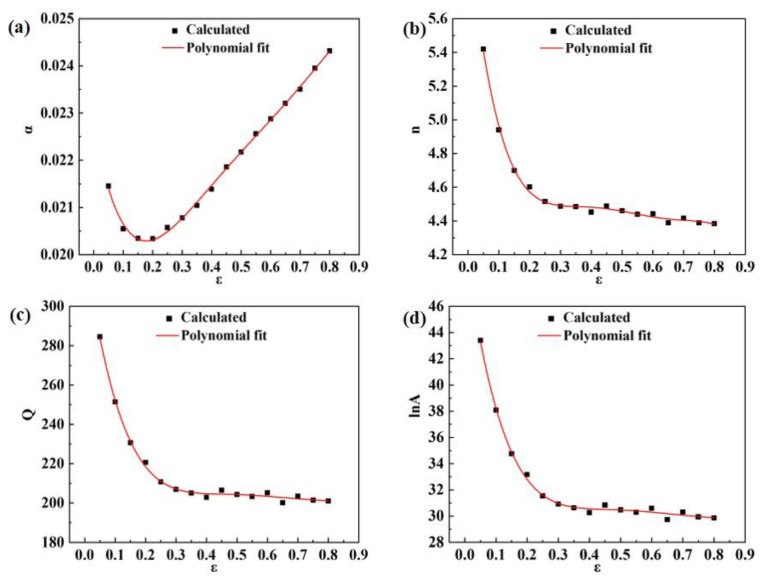
Relationship between material parameters and strain: (**a**) *α*, (**b**) *n*, (**c**) *Q*, (**d**) ln*A*.

The linear regression method can be used to find the values of material parameters such as *α*, *n*, *Q*, and ln*A* at different strains (0.05–0.8, with values taken at 0.05 intervals). In this experiment, as shown in Equation (9), a fifth-order polynomial function [15,16] is used for parameter fitting analysis, and the corresponding fitted curves are shown in Figure 5. The coefficients of the fitted material parameters such as *α*, *n*, *Q*, and ln*A* are shown in Table 3.

(9) $$\left\{\begin{array}{c} \alpha =B_{0}+B_{1}\varepsilon +B_{2}\varepsilon^{2}+B_{3}\varepsilon^{3}+B_{4}\varepsilon^{4}+B_{5}\varepsilon^{5}\\ n=C_{0}+C_{1}\varepsilon +C_{2}\varepsilon^{2}+C_{3}\varepsilon^{3}+C_{4}\varepsilon^{4}+C_{5}\varepsilon^{5}\\ Q=D_{0}+D_{1}\varepsilon +D_{2}\varepsilon^{2}+D_{3}\varepsilon^{3}+D_{4}\varepsilon^{4}+D_{5}\varepsilon^{5}\\ \ln A=E_{0}+E_{1}\varepsilon +E_{2}\varepsilon^{2}+E_{3}\varepsilon^{3}+E_{4}\varepsilon^{4}+E_{5}\varepsilon^{5} \end{array}\right.$$

The Arrhenius constitutive model of the homogenized 2195 Al-Li alloy considering strain compensation obtained by polynomial fitting of the material parameters can be expressed by Equation (9), which can be used to predict the flow stress for any given deformation condition.

(10) $$\sigma =\frac{1}{\alpha \left(\varepsilon \right)}\ln\left\{{\left(\frac{\dot{\varepsilon }\exp\left(Q\left(\varepsilon \right)/RT\right)}{A\left(\varepsilon \right)}\right)}^{1/n\left(\varepsilon \right)}+{\left[{\left(\frac{\dot{\varepsilon }\exp\left(Q\left(\varepsilon \right)/RT\right)}{A\left(\varepsilon \right)}\right)}^{2/n\left(\varepsilon \right)}+1\right]}^{1/2}\right\}$$

**Figure 6:**
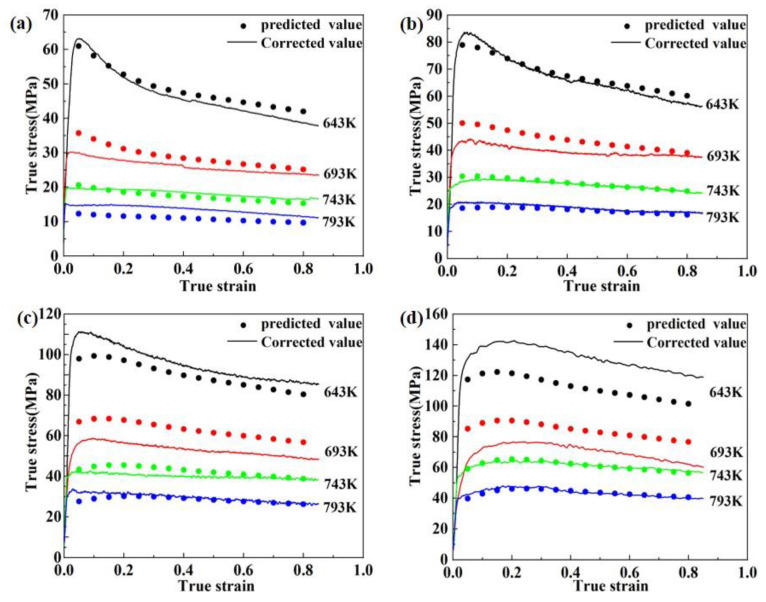
Comparison between the predicted results of flow stress and the corrected test results: (**a**) 0.001 s^−1^, (**b**) 0.01 s^−1^, (**c**) 0.1 s^−1^, (**d**) 1 s^−1^.

To investigate whether the established Arrhenius equation is consistent with the actual flow behavior of 2195 Al-Li alloy at different deformation temperatures and deformation rates, the flow stress values calculated by Equation (10) were compared with those obtained from actual hot compression tests, and the results are shown in Figure 6.

At high temperatures and low strain rates, the strain-compensated Arrhenius constitutive model fits the actual stress-strain curve more accurately, as seen in Figure 6. However, the degree of destabilization is increased at high strain rates (0.1~1 s^−1^) and deformation temperatures of 643–693 K. The predicted value is only 85.1% of the actual value, especially when the strain rate is 1 s^−1^ and the deformation temperature is 643 K. This implies that the flow stress behavior of 2195 Al-Li alloy cannot be well described by strain compensation correction alone.

The difference between the predicted and test stress values is quantified using the related coefficient (R) and average absolute relative error (AARE), which can be calculated using Equation (11) and Equation (12), respectively. R and AARE have computed values of 0.9758 and 9.51%, respectively. To further correct the Arrhenius constitutive model with strain compensation described previously, it is required to take into account the compensation of the flow stress by strain rate and deformation temperature.

(11) $$R=\frac{\sum_{i=1}^{N}\left(E_{i}-\bar{E}\right)\left(P_{i}-\bar{P}\right)}{\sqrt{\sum_{i=1}^{N}{\left(E_{i}-\bar{E}\right)}^{2}\sum_{i=1}^{N}{\left(P_{i}-\bar{P}\right)}^{2}}}$$

(12) $$AARE=\frac{1}{N}\sum_{i=1}^{N}\left|\frac{E_{i}-P_{i}}{E_{i}}\right|$$

#### 3.3.2. Arrhenius Constitutive Model Modified by Temperature and Strain Rate

**Figure 7:**
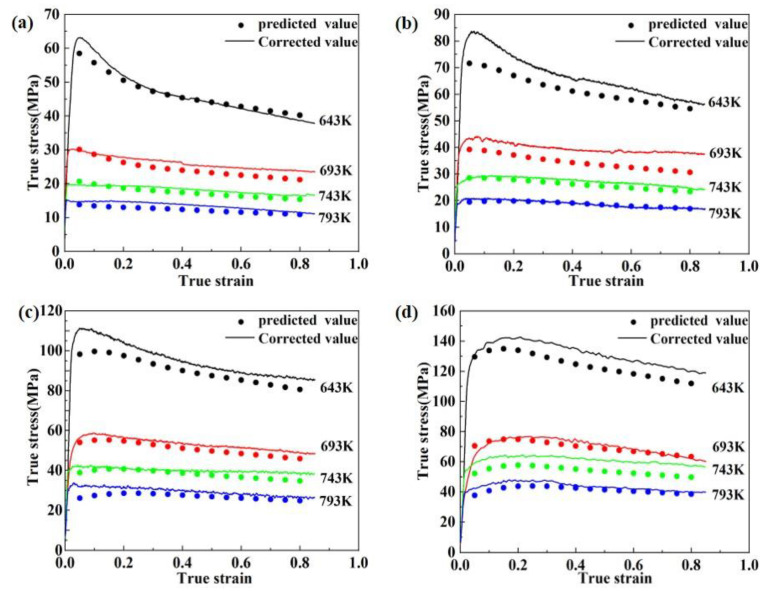
Comparison between the modified function results of flow stress and the corrected test results: (**a**) 0.001 s^−1^, (**b**) 0.01 s^−1^, (**c**) 0.1 s^−1^, (**d**) 1 s^−1^.

In this section, based on the already established Arrhenius constitutive equation considering strain compensation, a modified function for deformation temperature and strain rate is introduced, to establish an integrated constitutive model considering strain rate, deformation temperature and strain co-compensation, as shown in Equation (13). As indicated in Table 4, the modified function takes on a polynomial form, the test results obtained under various deformation situations are compared to the expected results first, and then the associated ratios are calculated. The ratio, which measures the difference between actual and predicted values, reflects how well the current constitutive model predicted values; the closer the ratio is to 1, the better. The data in Table 2 were then imported into Matlab software to establish different forms of polynomial functions, from which a function with high fitting accuracy and relatively simple structure was selected as the best-modified function in the form shown in Equation (14), which has a fitting accuracy of 0.9416 and the values of the correlation coefficients are shown in Table 5. A comparison of the correction values obtained from the Arrhenius constitutive model based on the strain rate, temperature and strain corrections with the flow stress values obtained from the actual hot compression tests is shown in Figure 7.

After calculation, the values of R and AARE are 0.9974 and 6.49%, respectively, which are significantly more accurate than the previous model. Therefore, the strain-compensated Arrhenius constitutive model considering strain rate and temperature corrections can accurately describe the flow behavior of 2195 Al-Li alloy.

(13) $$\sigma =f\left(\dot{\varepsilon },T\right)\cdot \frac{1}{\alpha \left(\varepsilon \right)}\ln\left\{{\left(\frac{\dot{\varepsilon }\exp\left(Q\left(\varepsilon \right)/RT\right)}{A\left(\varepsilon \right)}\right)}^{1/n\left(\varepsilon \right)}+{\left[{\left(\frac{\dot{\varepsilon }\exp\left(Q\left(\varepsilon \right)/RT\right)}{A\left(\varepsilon \right)}\right)}^{2/n\left(\varepsilon \right)}+1\right]}^{1/2}\right\}$$

(14) $$f\left(\dot{\varepsilon },T\right)=p_{0}+p_{1}\dot{\varepsilon }+p_{2}T+p_{3}{\dot{\varepsilon }}^{2}+p_{4}\dot{\varepsilon }\cdot T+p_{5}T^{2}+p_{6}{\dot{\varepsilon }}^{3}+p_{7}{\dot{\varepsilon }}^{2}\cdot T+p_{8}\dot{\varepsilon }\cdot T^{2}+p_{9}T^{3}$$

### 3.4. Processing Map and Microstructure

#### 3.4.1. Establishment of Processing Map

The processing map based on DMM, which comprises a power dissipation efficiency map and an instability map, and contains two portions of the safety domain and instability domain, can reflect the relationship between the microstructure evolution mechanism and the alloy’s thermal deformation parameters. During thermal deformation, the material can be regarded as an energy dissipater, and the total dissipated power *P* can be split into two parts: content G and co-content J. The strain rate sensitivity coefficient *m* can be used to express the relationship between temperature and strain rate under constant temperature and strain combinations.

(15) $$m=\frac{dJ}{dG}={\frac{d\ln\sigma }{d\ln\dot{\varepsilon }}}_{T,\varepsilon }$$

For an ideal linear dissipater, *m* = 1, and *J* obtains the maximum value *J*_max_. For a nonlinear dissipater, the power dissipation efficiency *η* can be expressed as the ratio of *J* to *J*_max_ as follows:

(16) $$\eta =\frac{J}{J_{\max}}=\frac{2m}{m+1}$$

However, a larger value of *η* does not mean better workability of the alloy, because the value of *η* under the conditions corresponding to the workability instability domain may also be larger [23,24]. Therefore, the instability criterion established by Prasad et al. [12] is used in this paper to determine the instability domain, as shown in Equation (17).

(17) $$\xi \left(\dot{\varepsilon }\right)=\frac{\partial \ln\left(\frac{m}{m+1}\right)}{\partial \ln\dot{\varepsilon }}+m<0$$

When *ξ* < 0, plastic flow destabilization will occur, that is, it is more likely to produce adiabatic shear bands, flow localizations and other microstructure defects during deformation.

**Figure 8:**
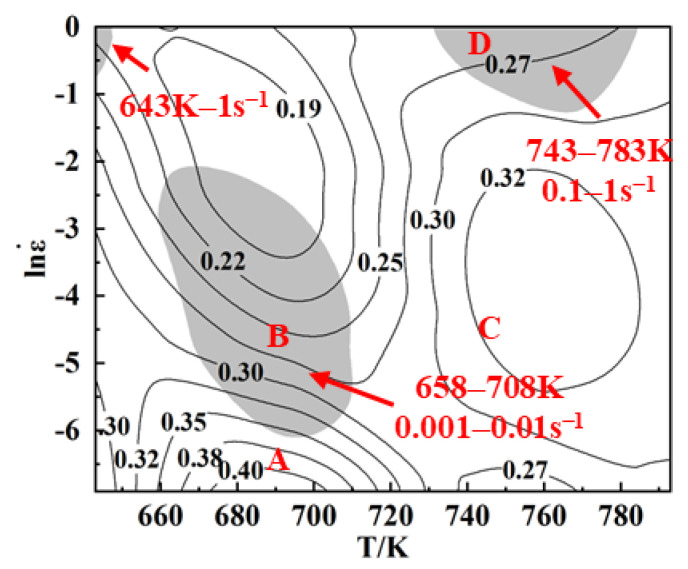
Processing map of the homogenized 2195 Al-Li alloy at the true strain of 0.8.

The processing map of the homogenized 2195 Al-Li alloy during hot compression with a strain of 0.8 was plotted in Figure 8, where the white and grey areas indicate the safety domains and the instability domains, respectively, and the numbers of the contour lines represent the values of the power dissipation coefficient *η*. It has been confirmed that the microstructure of the safety domains is mainly related to DRV, DRX and phase transition [25]. The higher *η* usually indicates more energy for microstructure evolution and better plastic deformation properties under the corresponding deformation conditions [26]. In general, the increase in the degree of DRX stimulates a raise in the power dissipation efficiency *η*.

The power dissipation exhibits peak areas in two temperature ranges: one is in the low-temperature range of 673–693 K, where the strain rate is below 0.001 s^−1^, and the other is in the high-temperature range of 743–793 K with a moderate strain rate of 0.01–0.1 s^−1^, as depicted in Figure 8. Additionally, a valley area of power dissipation is observed at high strain rates of 0.1–1 s^−1^ and medium temperatures of 673–703 K. Simultaneously, three instability domains are evident in the low-temperature, low-strain-rate region (658–708 K, 0.001–0.01 s^−1^), the low-temperature, high-strain-rate region (643 K, 1 s^−1^), and the high-temperature, high-strain-rate region (743–783 K, 0.3–1 s^−1^). The occurrence of the peak area of power dissipation at low temperature and low strain rate suggests the occurrence of DRV in the alloy. Meanwhile, the appearance of the peak area of power dissipation at high temperature and medium strain rate may be attributed to CDRX. Generally, alloys tend to undergo destabilization when deformed within the high-temperature and high-strain-rate range, consistent with the findings of Zhang et al. [14] and Wang et al. [16]. However, the alloy also exhibits destabilization at 658–708 K, 0.001–0.01 s^−1^, which requires verification through microstructural analysis.

Therefore, four typical regions were selected in the processing map; regions A (693 K, 0.001 s^−1^) and C (743 K, 0.01 s^−1^) are stable and regions B (693 K, 0.01 s^−1^) and D (743 K, 1 s^−1^) are unstable. The microstructure of these four regions was tested to analyze and discuss the deformation mechanism under different deformation conditions.

#### 3.4.2. Microstructure Characterization

**Figure 9:**
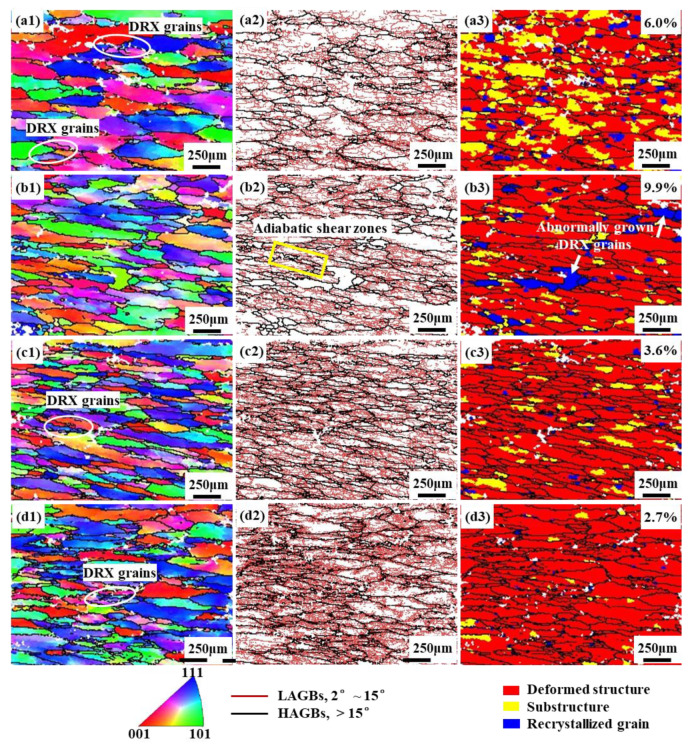
Inverse pole figures, grain boundary maps and recrystallization microstructure distributions of the alloy at (**a1**–**a3**) A (693 K, 0.001 s^−1^), (**b1**–**b3**) B (693 K, 0.01 s^−1^), (**c1**–**c3**) C (743 K, 0.01 s^−1^) and (**d1**–**d3**) D (793 K, 1 s^−1^).

Figure 9 illustrates the inverse pole figures, grain boundary maps and recrystallization microstructure distributions of the tested alloy under various strain rates and deformation temperatures. Thick black lines are used to indicate high angle grain boundaries (HAGBs, misorientation > 15°), and thin red lines are used to indicate low angle grain boundaries (LAGBs, misorientation 2–15°). In deformation microstructure distribution maps, red represents deformed structure (0–2°), yellow represents substructure (2–15°), and blue represents recrystallized grain (>15°).

As observed in Figure 9, a large number of original equiaxed grains are flattened and elongated along the compression direction, and some of the original flattened grain boundaries are serrated. Figure 9(a1,a2) reveal that in region A, where deformation occurs at a low temperature of 693 K and a low strain rate of 0.001 s^−1^, clear and uniformly distributed LAGBs can be observed inside the grains. This uniform plastic deformation indicates the occurrence of DRV in the alloy. The presence of partially recrystallized grains on the HAGBs, especially at the trigonal grain boundaries (as shown in the white elliptical box), with a significant difference in misorientation from the adjacent grains, indicates that DRX occurred under this condition [27], which is consistent with the results of the distribution of DRX grains demonstrated in Figure 9(a3), with a DRX degree of 6.0%. In addition, the power dissipation efficiency corresponding to this region in the processing map is also the largest, further confirming the alloy’s good workability. The microstructure of the material is displayed in Figure 9(b1–b3) when the strain rate reaches 0.01 s^−1^. It can be seen that the majority of the grains are distributed with uniform LAGBs inside, while only a small number of deformed grains with distinct grain boundary contours have almost no LAGBs inside. Moreover, adiabatic shear zones appear in specific locations (marked with yellow boxes). Figure 9(b3) reveals fewer recovery microstructures, more pronounced DRX (9.9%), significantly larger average grain size of recrystallized grains, and the presence of abnormally grown DRX grains (indicated by white arrows). These observations further support the notion that as the strain rate increases, the alloy deforms non-uniformly.

When the deformation condition is in the safety domain C, the power dissipation efficiency increases from 0.24 to 0.32. It can be seen from Figure 9(c1,c2) that the original grains undergo a more uniform plastic deformation and a large number of LAGBs are generated inside the grains. Figure 9(c3) shows the formation of fine chain-like recrystallized grains near the original grain boundaries, indicating that the recrystallization mechanism is dominated by DDRX under this deformation condition. In Figure 9(d1–d3), it can be seen that the alloy experiences more severe local plastic deformation when it is in the instability zone D. This can be attributed to the intensified local deformation resulting from higher strain rates and deformation temperatures. Consequently, the grains within the shear deformation zone become significantly elongated along the shear direction and exhibit diverse grain orientations. It is important to note that this condition exhibits the least amount of recrystallization (2.7%). The numerous small grains already formed near the original grain boundaries are not the result of recrystallization. Furthermore, the entanglement of numerous HAGBs suggests the challenges in coordinating material flow, indicating typical flow localization characteristics [28].

**Figure 10:**
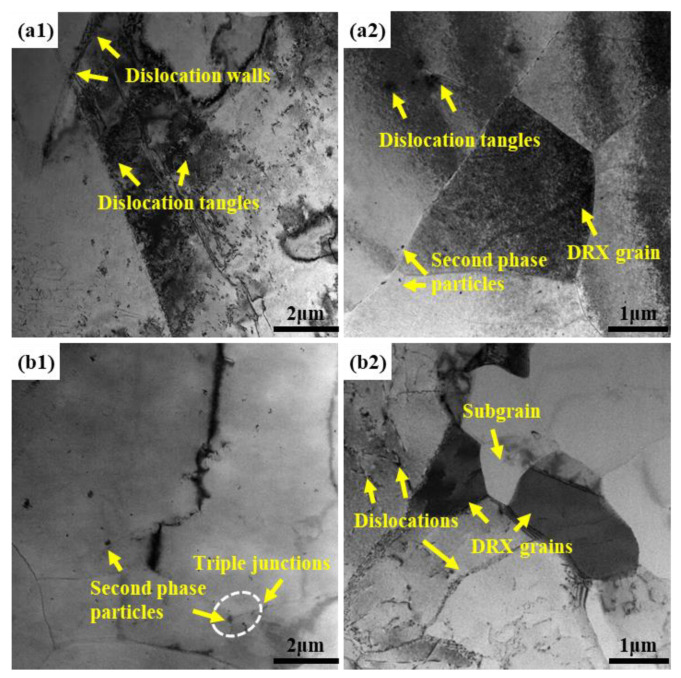
TEM images of the alloy under different deformation parameters: (**a1**,**a2**) region B, (**b1**,**b2**) region C.

The effects of deformation process parameters on dislocations and substructures were investigated using TEM, and the results are presented in Figure 10. Figure 10(a1,a2) clearly illustrates that the distribution of dislocations among different grains is non-uniform at 693 K and 0.01 s^−1^ (unstable zone B). Numerous dislocations accumulate at certain original HAGBs, forming a mass of dislocation walls and tangles. Conversely, there is no apparent presence of dislocations within another portion of the coarse grains, indicating the alloy’s instability under this deformation condition. Furthermore, the formation of DRX grain was observed near the original HAGB, which possess diameter larger than 2 μm. Additionally, a certain number of second phase particles exist at the grain boundaries, effectively pinning them and impeding dislocation movement. In the case of deformation occurring within the stable region C, as depicted in Figure 10(b1,b2), the number of dislocations and second phase particles within the matrix significantly decreases. The (sub)grain boundaries become more distinct, suggesting that higher temperatures promote dislocation climbing and cross-slip migration, as well as the dissolution of second phase particles. Furthermore, the generation of a greater number of subgrains and DRX grains with smaller sizes was observed. This observation demonstrates that most of the dislocations undergo DRV and DRX through rearrangement and annihilation [29], ultimately leading to a decrease in flow stress.

Considering the established processing map and the detected microstructure, the optimum deformation parameters of the homogenized 2195 Al-Li alloy were finally identified as 710–783 K and 0.001–0.12 s^−1^.

### 3.5. Application of Constitutive Model and Numerical Simulation Verification

This section describes the flow behavior of the material during thermal deformation using the established constitutive model. The model is then applied to the finite element simulation of 2195 Al-Li alloy extruded profiles with large shaped cross-sections in HyperXtrude software, and the extrusion parameters are shown in Table 1. Physical field quantities such as deformation behavior and stress distribution are analyzed to verify the accuracy and reliability of the constitutive model.

**Figure 11:**
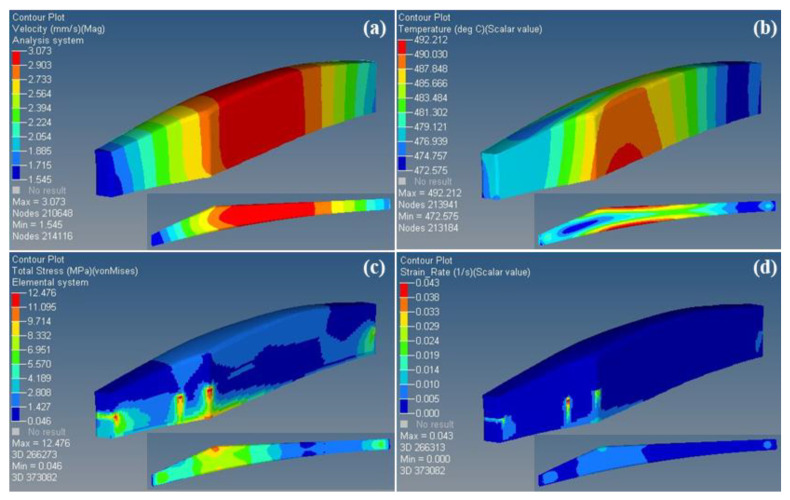
Numerical simulation results: (**a**) velocity field, (**b**) temperature field, (**c**) stress field, (**d**) strain rate field.

The results of the numerical simulation are presented in Figure 11. The small graph at the bottom right of each graph illustrates the distribution of the physical field of the profile section at the exit of the die. Figure 11a indicates that the velocity of the material is non-uniform along the extrusion direction and at the exit of the section. The velocity is significantly higher at the center than at the two ends, with the maximum velocity difference being 1.528 mm/s. Figure 11b shows that the profile reaches a maximum temperature of 765.2 K and a minimum temperature of 745.6 K during the extrusion process, with a maximum temperature difference of 19.6 K. The heat generated by the plastic deformation of the material itself, the friction between the ingot and the die, and the simultaneous heat transfer between the material and the outside lead to the temperature of the deformed material being higher than the initial extrusion temperature [30,31]. As shown in Figure 11c,d, the distribution of the equivalent stress and strain rate exhibit similar characteristics. The stress values on both surfaces at the thickest part of the profile are significantly higher than those at the left and right ends of the profile, reaching 12.48 MPa. This indicates that a stronger frictional effect occurs between the surfaces on both sides of the thickest part of the profile and the bearing of the die, which leads to the climbing of the stress values. Additionally, the profile’s irregular cross-section leads to different degrees of deformation in different areas, further increasing the unevenness of deformation.

In summary, the deformation temperature of the alloy ranges from 745 K to 765 K, and the deformation rate ranges from 0.000034 s^−1^ to 0.043 s^−1^. The range of value variation falls within the feasible region (710~783 K, 0.001~0.12 s^−1^) of the processing map established in Section 3.4. This indicates that the entire extrusion deformation occurs within the processing safety zone.

### 3.6. Confirmation Experiments

Practical extrusion tests were carried out on a 25 MN extruder to verify the accuracy of the constitutive model and numerical simulation, and the extruded profile of 2195 Al-Li alloy with high dimensional accuracy was successfully obtained, as shown in Figure 1b.

**Figure 12:**
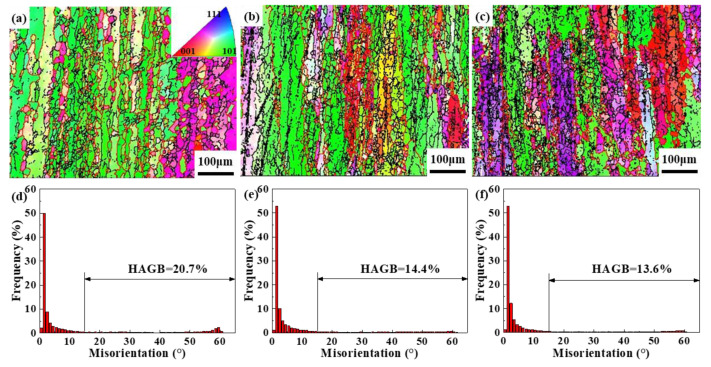
IPF maps and relative frequency of misorientation angles along the ED direction of the profile for different sampling areas: (**a**,**d**) P1, (**b**,**e**) P2, (**c**,**f**) P3.

To investigate the microstructure evolution during extrusion, EBSD analysis was performed on three observed surfaces of the profile, as shown in Figure 1c. Figure 12 illustrates the IPF maps and the relative frequency of misorientation angles at the three observation points. The deformed grains exhibit a fibrous and dispersed morphology along the extrusion direction (ED), as shown in Figure 12a–c. Notably, the grains in different locations display distinct preferred misorientations, specifically along the ED <001> and <101> crystal directions. Moreover, the microstructure does not display any significant signs of local plastic deformation or adiabatic shear bands, indicating that the chosen process parameters for extrusion fall within the safe processing zone.

Further investigation reveals the presence of smaller recrystallized grains exhibiting a chain distribution along the elongated grain boundary, with a small number of recrystallized grains also observed within the original grains, indicating that CDRX and DDRX occur concurrently during extrusion deformation. Figure 12d–f displays a substantial variation in the relative frequencies of misorientation angles among these three regions. The P1 region exhibits the highest proportion of HAGBs at 20.7%, while the P2 and P3 regions display lower proportions of HAGBs at 13.6% and 14.4%, respectively. Considering the physical field distribution in Figure 12, it can be inferred that the P1 region of the material experiences more severe plastic deformation and higher temperatures during the extrusion process, which may account for the increased generation of DRX grains in that region. During the continuous extrusion of 2195 Al-Li alloy, dislocations constantly propagate, with a majority of them climbing and slipping along the slip surface and shear zone. This process leads to the fragmentation of fibrous grains and the formation of numerous LAGBs [11,32]. Storage energy causes LAGBs to constantly rotate while absorbing dislocations, eventually resulting in the formation of recrystallized grains.

By utilizing the established multiparameter compensated constitutive model and processing map, the physical field distribution during the steady-state extrusion deformation stage of 2195 Al-Li alloy was accurately predicted. The microstructure analysis confirmed the production of high-quality extruded profiles. The successful fabrication of extruded profiles from 2195 Al-Li alloy serves as a compelling validation of the extensive applicability of accurate numerical simulations in industrial manufacturing. This approach not only reduces development costs and time but also enhances production efficiency and ensures stability in product quality.

## 4. Conclusions

In this study, the constitutive model and processing map of 2195 Al-Li alloy were established, and the accuracy of the constitutive model was proved by numerical simulation and practical extrusion test. The main conclusions are as follows:

1. An Arrhenius constitutive model considering deformation temperature, strain rate and strain co-compensation was established based on the true stress-strain data of homogenized 2195 Al-Li alloy after friction and temperature correction. This model significantly improved the accuracy of flow stress prediction during hot compression, with R and AARE values increasing to 0.9974 and 6.49%, respectively;
2. The processing safety zone and instability zone of 2195 Al-Li alloy were identified by employing the hot processing map. During hot compression, the alloy experienced both DRV and DRX. In the instability zone, considerable local plastic deformation bands and abnormally grown recrystallized grains were observed. The optimal deformation parameters for the 2195 Al-Li alloy were determined as 710~783 K and 0.001~0.12 s^−1^;
3. The physical field distribution of 2195 Al-Li alloy extruded profiles with large shaped cross-sections during extrusion was accurately predicted by a modified Arrhenius constitutive model. Practical extrusion tests produced extruded profiles with slight variations in microstructure in different regions. These variations were caused by uneven temperature and stress distributions, with higher temperature and more severe stress promoting the formation of HAGBs and DRX grains.

## Figures and Tables

**Table 1:** Process parameters for extrusion of 2195 Al-Li alloy.

Billet Size/mm	Container Diameter/mm	Billet Temperature/°C	Die Temperature/°C	Extrusion Ratio	Extrusion Velocity/(mm/s)
Ø496 × 600	Ø500	460	450	14.01	0.2

**Table 2:** Peak stress statistics.

Strain Rates/s^−1^	Temperature/K			
	643 K	693 K	743 K	793 K
0.001	63.17	30.04	19.80	15.33
0.01	83.72	44.18	29.46	20.92
0.1	111.25	58.81	42.65	33.74
1	142.30	76.78	64.64	48.04

**Table 3:** Coefficients of polynomial fitting material parameters.

ɑ		n		Q		lnA	
B0	0.02277	C0	6.12285	D0	330.50351	E0	50.77187
B1	−0.03474	C1	−17.64282	D1	−1103.31781	E1	−176.75073
B2	0.16345	C2	74.1133	D2	3799.09406	E2	607.48305
B3	−0.30791	C3	−150.3933	D3	−6394.61148	E3	−1021.01887
B4	0.27925	C4	146.24573	D4	5238.73818	E4	834.47043
B5	−0.09768	C5	−54.81721	D5	−1678.4414	E5	−266.50138

**Table 4:** The ratio of test stress values to predicted stress values at different strain rates and temperatures.

$\dot{\varepsilon }$	T	Experiment/Predicted	$\dot{\varepsilon }$	T	Experiment/Predicted
0.001	643	0.9635	0.1	643	1.0664
	693	0.9061		693	0.8502
	743	1.0495		743	0.9448
	793	1.2411		793	1.0341
0.01	643	0.9863	1	643	1.1753
	693	0.9044		693	0.8358
	743	0.9845		743	1.0009
	793	1.0568		793	1.0083

**Table 5:** Values of the coefficients in the modified function.

*p*_0_	*p*_1_	*p*_2_	*p*_3_	*p*_4_	*p*_5_	*p*_6_	*p*_7_	*p*_8_	*p*_9_
168.5	9.543	−0.6904	67.05	−3.203 × 10^−2^	9.43 × 10^−4^	−69.52	1.423 × 10^−2^	1.091 × 10^−5^	−4.269 × 10^−7^

## References

[1] Williams J.C., Starke E.A. (2003). Progress in structural materials for aerospace systems. Acta Mater..

[2] Araullo-Peters V., Gault B., Geuser F.D., Deschamps A., Cairney J.M. (2014). Microstructural evolution during ageing of Al–Cu–Li–x alloys. Acta Mater..

[3] Hales S.J., Hafley R.A. (1998). Texture and anisotropy in Al-Li alloy 2195 plate and near-net-shape extrusions. Mater. Sci. Eng. A.

[4] Jonas J., Sellars C., Tegart W. (1969). Strength and structure under hot-working conditions. Metall. Rev..

[5] Lin Y.C., Chen M.S., Zhong J. (2008). Constitutive modeling for elevated temperature flow behavior of 42CrMo steel. Comput. Mater. Sci..

[6] Lin Y.C., Ding Y., Chen M.S., Deng J. (2013). A new phenomenological constitutive model for hot tensile deformation behaviors of a typical Al–Cu–Mg alloy. Mater. Des..

[7] Zhao J.H., Deng Y.L., Tang J.G., Zhang J. (2019). Influence of strain rate on hot deformation behavior and recrystallization behavior under isothermal compression of Al-Zn-Mg-Cu alloy. J. Alloys Compd..

[8] Hu L., Lang M.W., Shi L.X., Li M.G., Zhou T., Bao C.L., Yang M.B. (2021). Study on hot deformation behavior of homogenized Mg-8.5Gd-4.5Y-0.8Zn-0.4Zr alloy using a combination of strain-compensated Arrhenius constitutive model and finite element simulation method. J. Magnes. Alloy..

[9] Xiao X., Liu G.Q., Hu B.F., Zheng X., Wang L.N., Chen S.J., Ullah A. (2012). A comparative study on Arrhenius-type constitutive equations and artificial neural network model to predict high-temperature deformation behaviour in 12Cr3WV steel. Comput. Mater. Sci..

[10] Meng Q.G., Bai C.G., Xu D.S. (2018). Flow behavior and processing map for hot deformation of ATI425 titanium alloy. J. Mater. Sci. Technol..

[11] Chen X.X., Zhao G.Q., Zhao X.T., Wang Y.X., Xu X., Zhang C.S. (2020). Constitutive modeling and microstructure characterization of 2196 Al-Li alloy in various hot deformation conditions. J. Manuf. Process..

[12] Prasad Y.V.R.K., Gegel H.L., Doraivelu S.M., Malas J.C., Morgan J.T., Lark K.A., Barker D.R. (1984). Modeling of dynamic material behavior in hot deformation: Forging of Ti-6242. Metall. Trans. A.

[13] Nayan N., Murty S.V.S.N., Chhangani S., Prakash A., Prasad M.J.N.V., Samajdar I. (2017). Effect of temperature and strain rate on hot deformation behavior and microstructure of Al-Cu-Li alloy. J. Alloys Compd..

[14] Zhang J.J., Yi Y.P., Huang S.Q., Mao X.C., He H.L., Tang J.G., Guo W.F., Dong F. (2021). Dynamic recrystallization mechanisms of 2195 aluminum alloy during medium/high temperature compression deformation. Mater. Sci. Eng. A.

[15] Yu W.C., Li H.Y., Du R., You W., Zhao M.C., Wang Z.A. (2018). Characteristic constitution model and microstructure of an Al-3.5Cu-1.5Li alloy subjected to thermal deformation. Mater. Charact..

[16] Wang Y.X., Zhao G.Q., Xu X., Chen X.X., Zhang C.S. (2019). Constitutive modeling, processing map establishment and microstructure analysis of spray deposited Al-Cu-Li alloy 2195. J. Alloys Compd..

[17] Dong Y.Y., Zhang C.S., Zhao G.Q., Guan Y.J., Gao A.J., Sun W.C. (2016). Constitutive equation and processing maps of an Al–Mg–Si aluminum alloy: Determination and application in simulating extrusion process of complex profiles. Mater. Des..

[18] Xu X., Ma X.W., Zhao G.Q., Chen X.X., Wang Y.X. (2021). Abnormal grain growth of 2196 Al-Cu-Li alloy weld seams during extrusion and heat treatment. J. Alloys Compd..

[19] Zhang C.S., Dong Y.Y., Wang C.X., Zhao G.Q., Chen L., Sun W.C. (2017). Evolution of transverse weld during porthole extrusion of AA7N01 hollow profile. J. Mater. Process. Technol..

[20] Ebrahimi R., Najafizadeh A. (2004). A new method for evaluation of friction in bulk metal forming. J. Mater. Process. Technol..

[21] Yang Q.B., Wang X.Z., Li X., Deng Z.H., Jia Z.H., Zhang Z.Q., Huang G.J., Liu Q. (2017). Hot deformation behavior and microstructure of AA2195 alloy under plane strain compression. Mater. Charact..

[22] Yang G., Xu W., Jin X., Wang Z., Shan D., Guo B. (2022). Hot deformation behavior and microstructure evolution of the spray deposited and secondary hot extruded 2195 Al–Li alloy. J. Mater. Res. Technol..

[23] Park S.Y., Kim W.J. (2016). Difference in the Hot Compressive Behavior and Processing Maps between the As-cast and Homogenized Al-Zn-Mg-Cu (7075) Alloys. J. Mater. Sci. Technol..

[24] Wu H., Wen S.P., Huang H., Gao K.Y., Wu X.L., Wang W., Nie Z.R. (2016). Hot deformation behavior and processing map of a new type Al-Zn-Mg-Er-Zr alloy. J. Alloys Compd..

[25] Miao J.S., Sutton S., Luo A.A. (2022). Deformation microstructure and thermomechanical processing maps of homogenized AA2070 aluminum alloy. Mater. Sci. Eng. A.

[26] El Mehtedi M., Gabrielli F., Spigarelli S. (2014). Hot workability in process modeling of a bearing steel by using combined constitutive equations and dynamic material model. Mater. Des..

[27] Shen B., Deng L., Wang X.Y. (2015). A new dynamic recrystallisation model of an extruded Al-Cu-Li alloy during high-temperature deformation. Mater. Sci. Eng. A.

[28] Lin Y.C., Li L.T., Xia Y.C., Jiang Y.Q. (2013). Hot deformation and processing map of a typical Al–Zn–Mg–Cu alloy. J. Alloys Compd..

[29] Wang Z.Y., Zhang K.S., Song Y.Q., Ali R.A., Chen W.L., Wang X.X. (2022). Constitutive behavior and microstructural evolution of 2060 Al–Li alloy under high strain rate: Experiment and simulation. Mater. Sci. Eng. A.

[30] Attarilar S., Gode C., Mashhuriazar M.H., Ebrahimi M. (2021). Tailoring twist extrusion process; the better strain behavior at the lower required loads. J. Alloys Compd..

[31] Yi J., Wang Z.H., Liu Z.W., Zhang J.M., He X. (2018). FE analysis of extrusion defect and optimization of metal flow in porthole die for complex hollow aluminum profile. Trans. Nonferrous Met. Soc. China.

[32] Jiang J.F., Wang Y., Liu Y.Z., Xiao G.F., Li H. (2021). Microstructure and mechanical properties of 7005 aluminum alloy processed by one-pass equal channel reciprocating extrusion. Trans. Nonferrous Met. Soc. China.

